# The Diagnostic Dilemma of an Odontogenic Jaw Lesion in a Pediatric Patient: A Case Report

**DOI:** 10.7759/cureus.31225

**Published:** 2022-11-08

**Authors:** Rutuja Ragit, Punit Fulzele, Nilima R Thosar, Monika Khubchandani

**Affiliations:** 1 Pediatric and Preventive Dentistry, Sharad Pawar Dental College & Hospital, Datta Meghe Institute of Medical Sciences, Wardha, IND; 2 Pediatric Dentistry, Sharad Pawar Dental College & Hospital, Datta Meghe Institute of Medical Sciences, Wardha, IND

**Keywords:** histopathologic diagnosis, marsupialization, dentigerous cyst, radicular cyst, ameloblastoma

## Abstract

This paper presents a case report of a 10-year-old child patient reported with the chief complaint of a painless, hard swelling in the lower right back region of the jaw. The clinical and radiographic examination, including intraoral periapical radiograph (IOPA) and cone-beam CT (CBCT), was performed. Conservative treatment was planned based on the clinical and radiological diagnosis of the cyst. But the histological examination revealed unicystic ameloblastoma (UA). This clinical case of UA, which was misdiagnosed as a combination of a radicular cyst and a dentigerous cyst, is being presented to highlight the importance of histopathologic investigation of all tissue specimens retrieved after surgery, particularly when the clinical and radiological findings are insignificant.

## Introduction

Odontogenic and non-odontogenic tumors of the maxillofacial skeleton can be malignant or benign. Ameloblastoma is considered to be the most common of all tumors of odontogenic origin [1]. Ameloblastomas are benign odontogenic tumors with a rare ability to metastasize because of their frequently recurring, locally aggressive, infiltrative nature. Ameloblastoma comprises 10% of odontogenic tumors. Ameloblastomas are rare in the pediatric patient population and often develop between the third and fourth decades, making up about 10% to 15% of all documented occurrences. Unicystic ameloblastoma (UA) is a rare type of ameloblastoma that typically affects younger patients [2]. Ameloblastomas are categorized into three types by the World Health Organization: conventional, peripheral, and unicystic. UA is an odontogenic tumor that exhibits clinical, radiological, or gross characteristics of a mandibular cyst. However, upon histologic inspection, a typical ameloblastomatous epithelium lining a portion of the cyst cavity is seen, with or without mural and luminal tumor development [3]. According to Kramer, a cyst is "a pathological cavity with fluid, semifluid, or gaseous contents and which is not formed by the accumulation of pus" [4]. With very few exceptions, bone cysts lined with epithelium are only present in the jaws. One of the most frequent lesions seen in the dental office is an odontogenic cyst [5]. Dental cysts can be of noninflammatory origin (dentigerous cysts) or inflammatory origin (radicular cysts). Dentigerous cysts were found to be the most prevalent type, accounting for 45.6% of developing cysts, followed by odontogenic keratocysts, which account for 5.6%, and among cysts of inflammatory origin, the most common variety was determined to be radicular cysts, i.e., 31.2% [6]. Radicular cysts are the most prevalent cystic lesions and a frequent source of long-term jaw swellings. They are uncommon in the primary dentition and are caused by inflammation that follows pulpal necrosis and arises from epithelial remnants of the periodontal ligament. The most frequent cause of a radicular cyst is dental caries related to a deciduous molar. The cyst of odontogenic origin that encircles the crown of an unerupted tooth is known as a dentigerous cyst because of the fluid accumulation between the unerupted tooth crown and the epithelium. The most commonly impacted teeth are maxillary and mandibular third molars and canine. Therefore, these cysts occur most commonly in this region [7]. In this case report, a pediatric patient presented with a large UA in the body of the mandible. The radiographic and clinical findings suggested a combination of a radicular cyst with a decayed first primary molar and a dentigerous cyst encircling an unerupted first premolar. However, the histopathological finding revealed ameloblastoma.

## Case presentation

**Figure 1:**
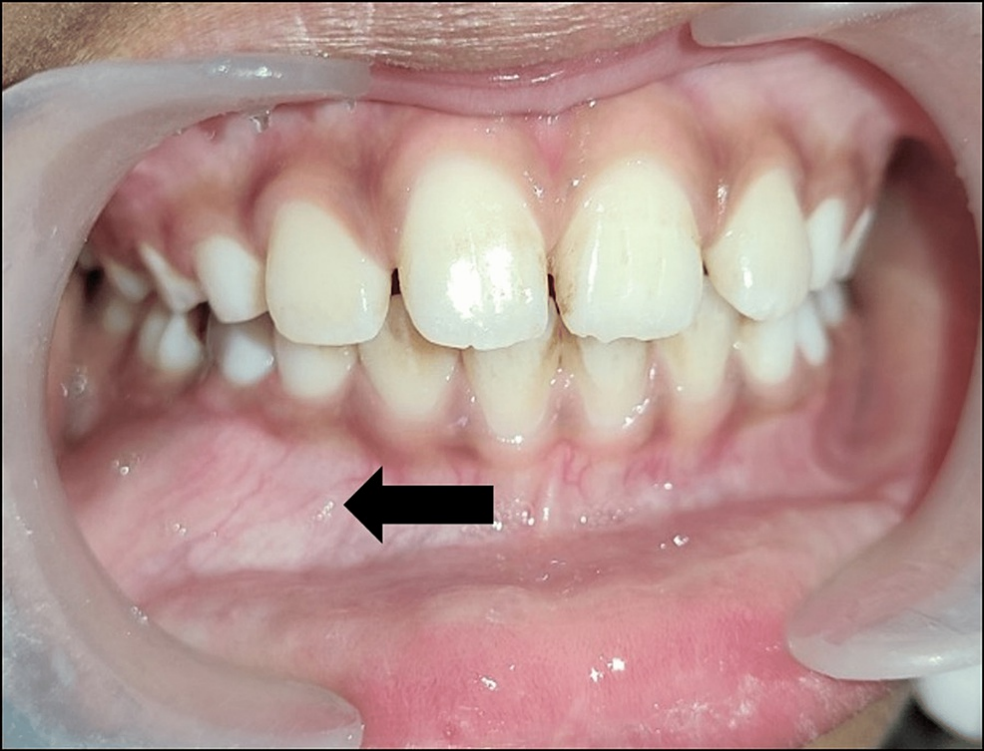
Intraoral examination showing a poorly demarcated expansile swelling involving the right posterior mandibular region.

A 10-year-old boy was reported to the Department of Pediatric and Preventive Dentistry with the chief complaint of a hard swelling that was painless and present for one month in the lower right back area of the jaw. There was no accompanying fever. On palpation, a well-defined firm external swelling was seen on the mandibular lower body, which was hard in consistency. The overlying skin of the swelling was of standard color, consistency, and temperature. The intraoral examination showed a hard, firm swelling extending from the first primary molar to the second primary molar along the buccal vestibule. The bone margins were blended imperceptibly into the surrounding bone (Figure 1).

**Figure 2:**
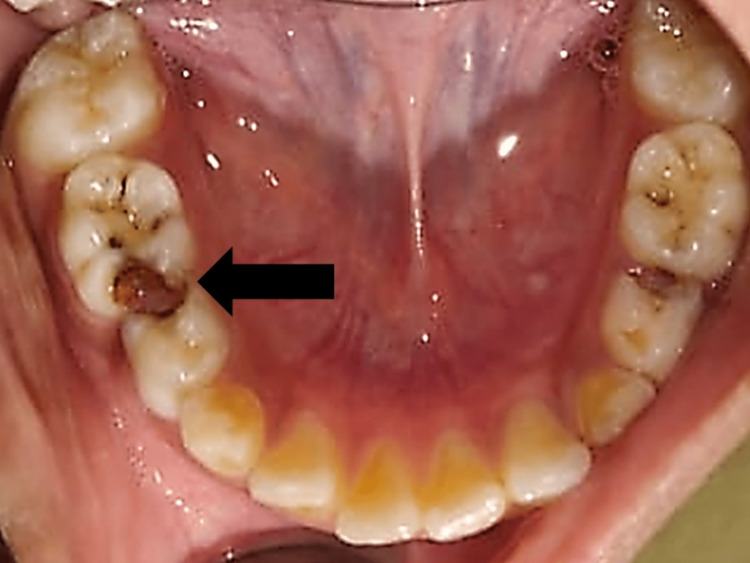
Intraoral image showing distoproximal caries with 84 and mesial proximal caries with 85.

The right submandibular lymph nodes presented tender lymphadenopathy. There was unrestricted mouth opening. A mild lingual and buccal cortical expansion was seen. The first primary molar (84) was deeply carious near the swelling area. Proximal caries were also seen with the second primary molar (85; Figure 2). No mobility of associated teeth was seen. When the surrounding soft tissue of the first primary molar was palpated, the patient complained of pain, but no additional symptoms of inflammation were found.

**Figure 3:**
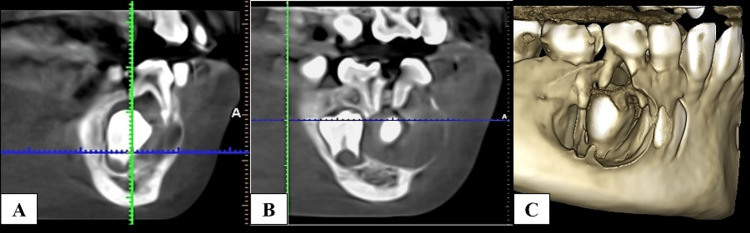
CBCT images: (A) an expansile unilocular lesion involving the posterior part of the body of the mandible; (B) the bilateral cortical plate expansion and the second primary molar (45) was unusually pushed into an ectopic position within the radiolucency distally; (C) the involvement of mesial root of the second primary molar (85) within the lesion. CBCT, cone-beam CT

 A cone-beam CT (CBCT) scan was suggested to the patient. The complete extent of this lesion was seen on CT scans, which also demonstrated no extension into the soft tissue around it. The scan showed an expansile lesion with dimensions 17.2 mm × 14.8 mm × 14.0 mm, which caused the buccal and lingual cortical plates to expand (Figure 3A, 3B, 3C).

**Figure 4:**
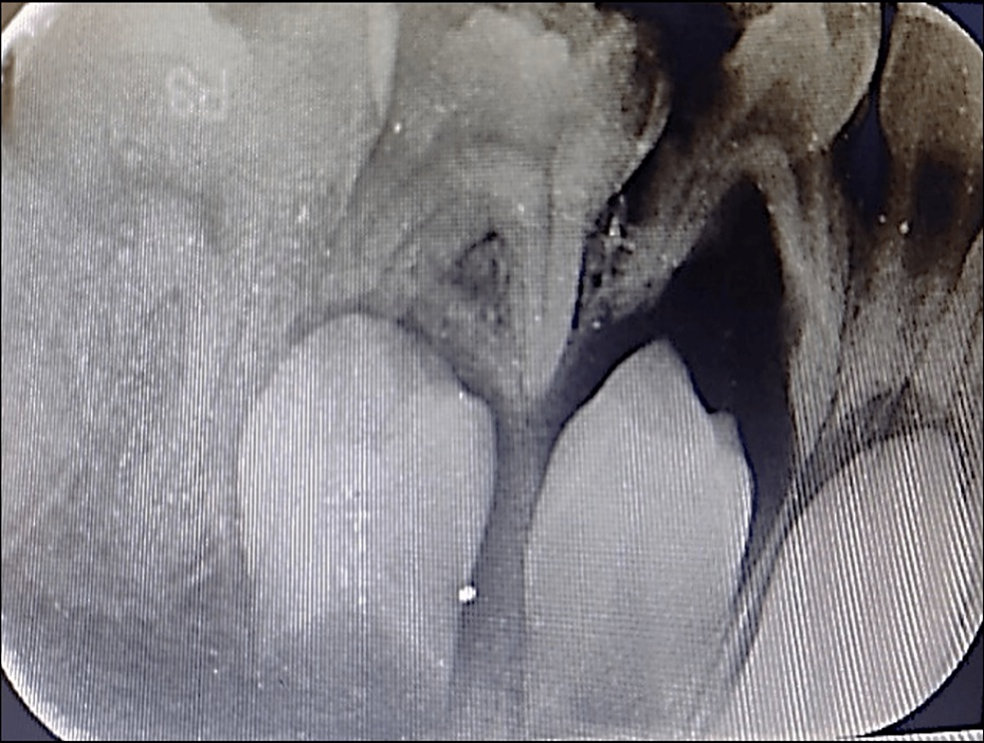
The IOPA radiograph revealing a periapical radiolucent lesion extending from the distal root of 83 to the mesial root of 85. IOPA, intraoral periapical

The intraoral periapical (IOPA) radiograph revealed an extended radiolucent periapical lesion from the distal root of 83 to the mesial root of 85 (Figure 4). Pulpal involvement of the first primary molar and the mesial root resorption of the second primary molar was seen. Around the roots of the primary molars, there was a noticeable, significant radiolucency. The radiograph also revealed that the second primary molar (45) was unusually pushed into an ectopic position within the radiolucency distally.

Fine needle aspiration cytology (FNAC) revealed fluid with a pale straw color. There was the possibility of a cyst, and marsupialization was planned. Treatment was scheduled under local anesthesia (LA) because the patient was cooperative.

Treatment

**Figure 5:**
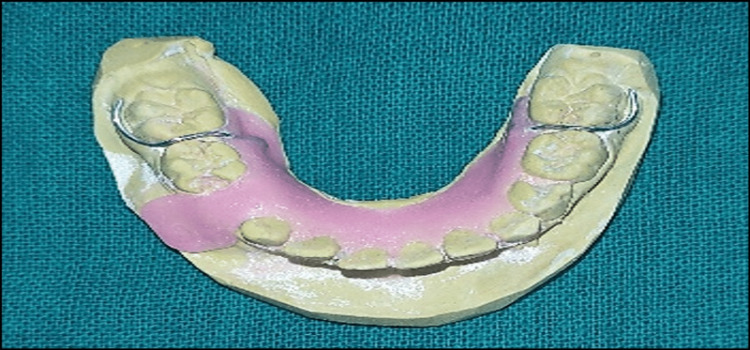
An acrylic splint fabricated on a cast.

The clinical and radiographic examination showed a possibility of the following differential diagnosis: 1) radicular cysts, 2) dentigerous cysts, and 3) odontogenic keratocysts. The suggested treatment for a dentigerous or radicular cyst in a child is marsupialization, which preserves the permanent tooth and allows it to erupt. A conservative treatment was intended to save the premolar tooth bud. Before the extraction, the impression was taken for fabricating an acrylic splint (Figure 5).

**Figure 6:**
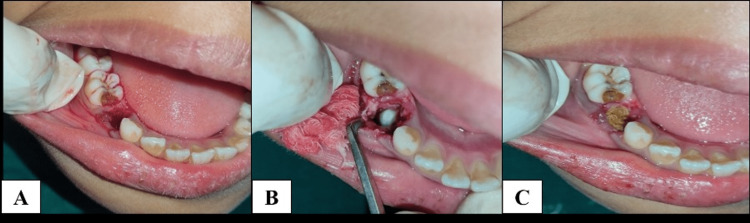
Intraoperative images: (A) extraction of 84 followed by marsupialization, (B) irrigation of the defective region with a saline and betadine solution, and (C) placement of a betadine-soaked pack inside the cavity for three days.

After the administration of LA, the extraction of 84 was done, followed by marsupialization. The region was irrigated with a saline and betadine solution. A betadine-soaked pack was placed inside the cavity for three days (Figure 6A, 6B, 6C).

**Figure 7:**
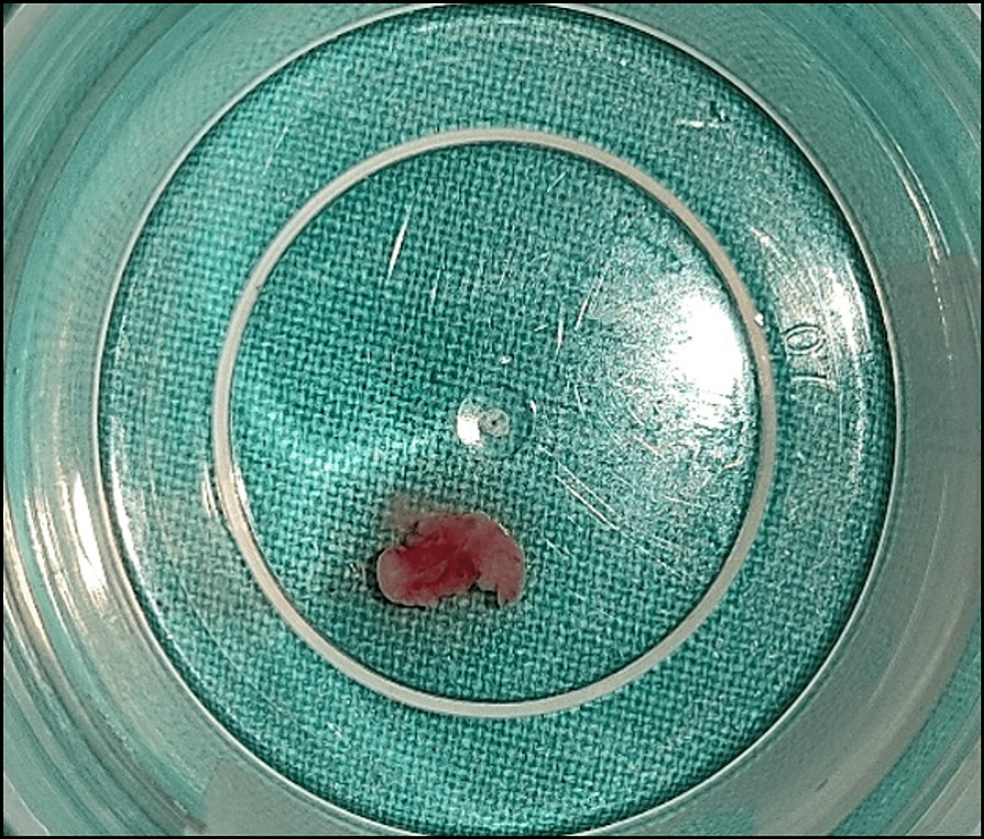
Excised tissue sample.

The excised tissue samples were sent in two different bottles for histopathological analysis. The first bottle contained the extracted tooth (84), and the second bottle contained the soft tissue lining of the lesion (Figure 7).

**Figure 8:**
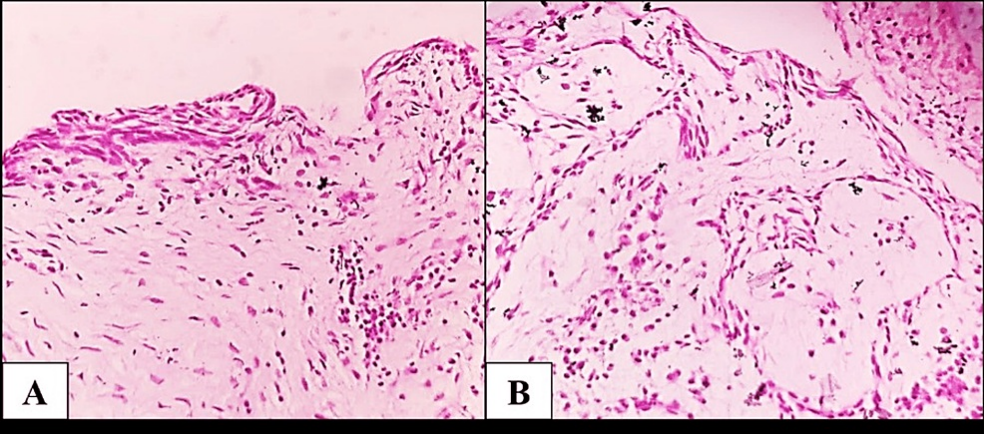
Histopathological investigations: (A) a cystic space lined by odontogenic epithelium with hyperchromatic nuclei and (B) luminal proliferation of odontogenic cells suggestive of a plexiform pattern.

Histopathological analysis of the lesional tissue reveals a connective tissue wall made of fibrocellular connective tissue, a cystic space lined by odontogenic epithelium with hyperchromatic nuclei, and suprabasal cells that resemble stellate reticulum-like cells (Figure 8A). The image displays odontogenic cells proliferating luminally in a way that suggests a plexiform pattern (Figure 8B). Based on the histology report, the diagnosis of unicystic plexiform ameloblastoma was confirmed.

**Figure 9:**
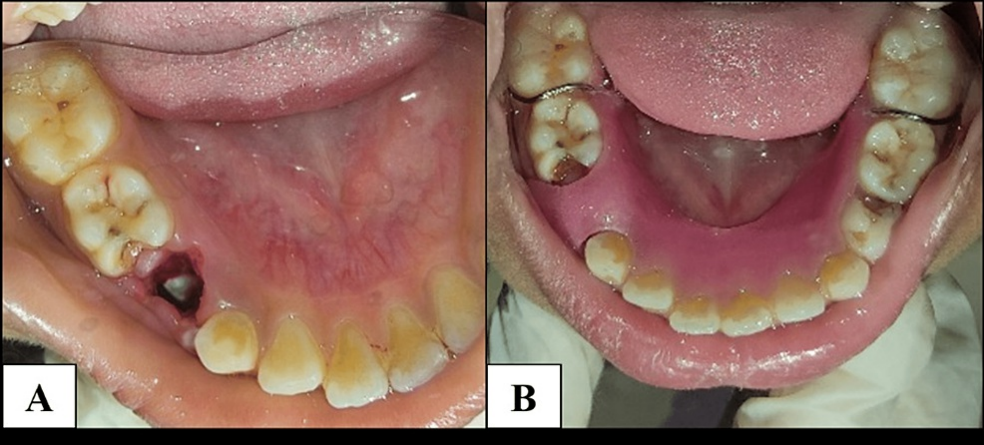
An acrylic splint served as an obturator for a defective cavity formed after treatment.

After three days, the patient was recalled, the betadine dressing was removed, and an acrylic splint was given (Figure 9). This acrylic splint served as an obturator and allowed complete healing of the lesion cavity.

**Figure 10:**
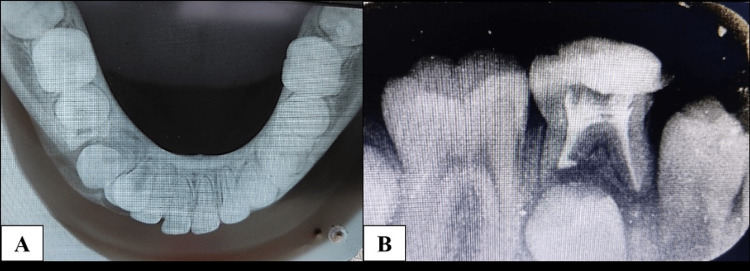
Occlusal and IOPA radiographs: (A) occlusal radiograph showing reduced buccal and lingual cortical plate expansion and (B) IOPA indicating pulpectomy with 85 (radiograph taken after obturation and before final restoration) and erupting the first premolar (44). IOPA, intraoral periapical

Access opening was done with 85, followed by metrogyl irrigation, as there were deep proximal caries and lesions, including a mesial root of 85, which was partially resorbed. Rather than extraction, tooth 85 was preserved to help guide the eruption of the permanent second premolar, i.e., 45. Three months later, it was found that the underlying permanent mandibular premolar 44 partially erupted into the position they usually occupy in the oral cavity, and healing was seen in the cystic lesion space. Therefore, the splint was removed. Slightly expanded buccal cortical bone was seen (Figure 10A). Pulpectomy with 85 was completed after three months as the patient failed to report to the department in this period of three months, and there was no presence of pain and mobility (Figure 10B). The patient is still on regular periodic evaluation.

## Discussion

Robinson and Martinez first used the term *unicystic ameloblastoma* in 1977 [8]. UA is a term used for cystic unilocular lesions that exhibit the radiologic, clinical, or gross characteristics of a cyst of the mandible but, on histologic analysis, showed the characteristic epithelium lining of ameloblastoma, either with or without mural or luminal tumor development in the cyst cavity. Among all intraosseous ameloblastomas, UA occurs in 5% to 10% of the cases and tends to be less aggressive and respond better to conservative surgery than solid or multicystic ameloblastomas [3,8]. Ameloblastomas are categorized into conventional, extra-osseous, unicystic, peripheral, and metastasizing types [8]. UA is further subclassified as follows by Ackerman et al.: type I (luminal or simple), in which the confinement of the tumor is to the cyst epithelial lining; type II (intraluminal), in which the neoplastic epithelium with nodular proliferation protrudes into the lumen; and type III (intramural or mural), in which plexiform or follicular pattern of ameloblastomatous epithelium invades the wall of connective tissue [9]. This classification is believed to directly affect the biological behavior, care, and prognosis of UAs. The most frequently degrading jaw lesions are odontogenic cysts, divided into developing and inflammatory types based on their cause. Radicular cysts are inflammatory and the most commonly occurring odontogenic cysts, whereas in developmental cysts, the most common type is dentigerous cysts [7]. In the present case, the radiologic investigation suggested a combination of the radicular cyst with a decayed first primary molar and the dentigerous cyst with the first premolar. Additionally, the aspirate collected during the initial clinical examination revealed inflammatory cells in the cystic fluid on cytological analysis. For radicular cysts, the most suitable treatment options include total enucleation in small lesions cases and marsupialization in larger cysts cases or a combination of the two techniques [7]. Marsupialization is the suggested treatment for dentigerous cysts in youngsters to preserve and give a chance for the permanent tooth to erupt. According to some authors, definitive therapy includes the extraction of the associated tooth and enucleation of the pericoronal soft tissue [6]. The marsupialization method was ultimately chosen as the preferred treatment option by considering the patient's age and clinical and radiologic characteristics as it results in the least amount of patient morbidity and has the most negligible impact on quality of life. The main drawback of marsupialization is that pathologic tissue is not thoroughly examined histologically and is instead left in place. Cyst enucleation combined with the affected tooth is the gold standard in cases of long-standing, larger lesions and unerupted teeth positioned unfavorably [7]. The histopathologic analysis of the tissue sample in the present case revealed ameloblastic alterations that were restricted to the cyst's luminal surface, and the diagnosis of unicystic plexiform ameloblastoma was confirmed. UA recurrence depends on the histologic subtypes; those that invade the fibrous wall occur at a rate of 35.7%, whereas others occur at just 6.7% [10]. As UAs are thought to be a less aggressive variety of ameloblastomas, they can be successfully eliminated with simple enucleation or less invasive surgery [3]. According to Lau and Samman's systematic review, resection had the lowest recurrence rate (3.6%) for treating UAs [11]. Although resection for UA has a high success rate, more conservative therapy is typically preferred to decrease morbidity and improve quality of life. Although simple enucleation may not be necessary, UA is less aggressive than its solid equivalent and should respond to less severe treatment techniques. The highest recurrence rate of 30.5% was seen when enucleation was used exclusively. Pogrel and Montes concluded that intra-osseous ameloblastomas could not be managed with simple enucleation alone because it has a high recurrence rate of up to 60% [12]. A recurrence rate of 16% was observed when enucleation followed by the use of Carnoy's solution was done [11]. For UA treated by marsupialization plus additional surgical operations (resection or enucleation), the recurrence incidence was 18%. As the average time for recurrences has been observed to be around five years, it is recommended that all patients have long-term follow-ups [13,14]. In similar cases, marsupialization has several benefits, including a reduction in the cavity size, a reduction in injury to the affected bone tissue, stimulation of osteogenesis, reduction in the chance of injury to neighboring anatomical structures such as the maxillary sinus and inferior alveolar nerve, and, lastly, promotion of the eruption of the concerned teeth [15]. On the other hand, there are drawbacks to this treatment option, such as the requirement for good cooperation by patients and the need for follow-ups for a long period until the eruption of the involved tooth, both of which may not be possible for many patients. These drawbacks may be the reason for marsupialization failure as a treatment modality. In the current case, the histopathologic examination made it possible to make a correct diagnosis. Therefore, a histopathologic examination is necessary for a correct diagnosis.

## Conclusions

Based on the clinical and radiological diagnoses of the cyst, in this case, conservative treatment options, such as marsupialization rather than enucleation, are being considered to preserve the tooth bud of the premolar and enable tooth eruption. However, to rule out potential ameloblastomatous alterations, we conclude that the surgical protocol must include the postoperative histopathologic assessment for all lesions, adequate monitoring of the patient, and addressing of any recurrences.
